# HSPB2 Is Dispensable for the Cardiac Hypertrophic Response but Reduces Mitochondrial Energetics following Pressure Overload In Mice

**DOI:** 10.1371/journal.pone.0042118

**Published:** 2012-08-01

**Authors:** Takahiro Ishiwata, András Orosz, Xiaohui Wang, Soumyajit Banerjee Mustafi, Gregory W. Pratt, Elisabeth S. Christians, Sihem Boudina, E. Dale Abel, Ivor J. Benjamin

**Affiliations:** 1 Laboratory of Cardiac Disease, Redox Signaling and Cell Regeneration, Division of Cardiology, University of Utah School of Medicine, Salt Lake City, Utah, United States of America; 2 Division of Endocrinology, Metabolism and Diabetes, and Program in Molecular Medicine, University of Utah School of Medicine, Salt Lake City, Utah, United States of America; 3 Department of Biochemistry, University of Utah, School of Medicine, Salt Lake City, Utah, United States of America; Univ. Med. Center Groningen, Univ. of Groningen, Netherlands

## Abstract

**Background:**

CryAB (HspB5) and HspB2, two small heat shock genes located adjacently in the vertebrate genome, are hypothesized to play distinct roles. Mice lacking both *cryab* and *hspb2* (DKO) are viable and exhibit adult-onset degeneration of skeletal muscle but confounding results from independent groups were reported for cardiac responses to different stressful conditions (i.e., ischemia/reperfusion or pressure overload). To determine the specific requirements of HSPB2 in heart, we generated cardiac-specific HSPB2 deficient (HSPB2cKO) mice and examined their cardiac function under basal conditions and following cardiac pressure overload.

**Methodology/Principal Findings:**

Transverse aortic constriction (TAC) or sham surgery was performed in HSPB2cKO mice and their littermates (HSPB2wt mice). Eight weeks after TAC, we found that expression of several small HSPs (HSPB2, 5, 6) was not markedly modified in HSPB2wt mice. Both cardiac function and the hypertrophic response remained similar in HSPB2cKO and HSPB2wt hearts. In addition, mitochondrial respiration and ATP production assays demonstrated that the absence of HSPB2 did not change mitochondrial metabolism in basal conditions. However, fatty acid supported state 3 respiration rate (ADP stimulated) in TAC operated HSPB2cKO hearts was significantly reduced in compared with TAC operated HSPB2wt mice (10.5±2.2 vs. 12.8±2.5 nmol O_2_/min/mg dry fiber weight, P<0.05), and ATP production in HSPB2cKO hearts was significantly reduced in TAC compared with sham operated mice (29.8±0.2 vs. 21.1±1.8 nmol ATP/min/mg dry fiber weight, P<0.05). Although HSPB2 was not associated with mitochondria under cardiac stress, absence of HSPB2 led to changes in transcript levels of several metabolic and mitochondrial regulator genes.

**Conclusions/Significance:**

The present study indicates that HSPB2 can be replaced by other members of the multigene small HSP family under basal conditions while HSPB2 is implicated in the regulation of metabolic/mitochondrial function under cardiac stress such pressure overload.

## Introduction

The small MW heat shock proteins (i.e. sHSPs, approximately 15–30 kDa) are expressed in virtually all organisms, from bacteria to humans. They are evolutionarily related via a conserved sequence domain in the carboxyl region, termed the α-crystallin domain [1002C2]. Functionally, most sHSPs display chaperone-like activity to maintain misfolded proteins in soluble but inactive states and, furthermore, protect cells against stressful conditions. While the selective patterns of expression suggest that sHSPs may impart tissue-specific and specialized roles, the nature of these functions is still under active investigation [3]

The small HSPs αB-crystallin (*cryab* also named *hspb5*) and *hspb2* form a bidirectional gene pair that reside on chromosome 9 and chromosome 11 in mouse and human genomes, respectively [4]. *Cryab* and *hspb2* are the result of a gene duplication event and share a common promoter region, although *cryab*, but not *hspb2*, is stress-inducible [4], [5]. Their different intracellular distribution and interactions with other sHSPs suggest that they have distinct intracellular functions [6], [7]. Due to their adjacent genomic organization, initial gene targeting strategy inadvertently resulted in simultaneous *cryab* and *hspb2* being deleted in mice [8]. The resulting double knock-out (DKO) mice have been extensively characterized and investigated with respect to the dual roles of HSPB2 and CRYAB during ischemia, oxidative stress [9], [10], [11], [12] as well as in response to pressure overload conditions [13], [14]. DKO showed more severe hypertrophic response against pressure overload while transgenic overexpression of CRYAB attenuated the hypertrophic response [13], [14]

HSPB2 is expressed at high level in skeletal muscle and the heart [15]. Previous work showed that overexpressed HSPB2 co-localizes with the outer mitochondrial membrane after stress [16], suggesting that HSPB2 would be related to the mitochondrial-dependent cell protection/death pathways and/or the mitochondrial bioenergetic pathways. It has been reported that mitochondrial permeability transition and calcium uptake were increased in DKO cardiomyocytes and mitochondrial respiration rate using skinned fibers from DKO myocardium were reduced compared with wild type(WT) [10], [17].

Therefore, beyond the DKO model affecting both CRYAB and HSPB2 expression, a new mouse model that targets specifically hspb2 is required to determine the distinct tissue-specific functions of HSPB2 *in vivo.* The present study reports the creation of a conditional floxed hspb2 allele and the production of mice with a cardiac-specific knockout of *hspb2* (HSPB2cKO). Our data reveal that the absence of HSPB2 in the heart does not significantly affect the cardiac hypertrophic response to pressure overload stimuli, but that HSPB2 deficiency depresses mitochondrial fatty acid beta-oxidation and ATP production under these conditions

## Materials and Methods

### Experimental Animals

These studies were approved by the Animal Care and Use Committees of the University of Utah and adhered to the Guide for the Care and Use of Laboratory Animals (NIH).

### Generation of hspb2 Conditional Knock-out Mice

**Figure 1:**
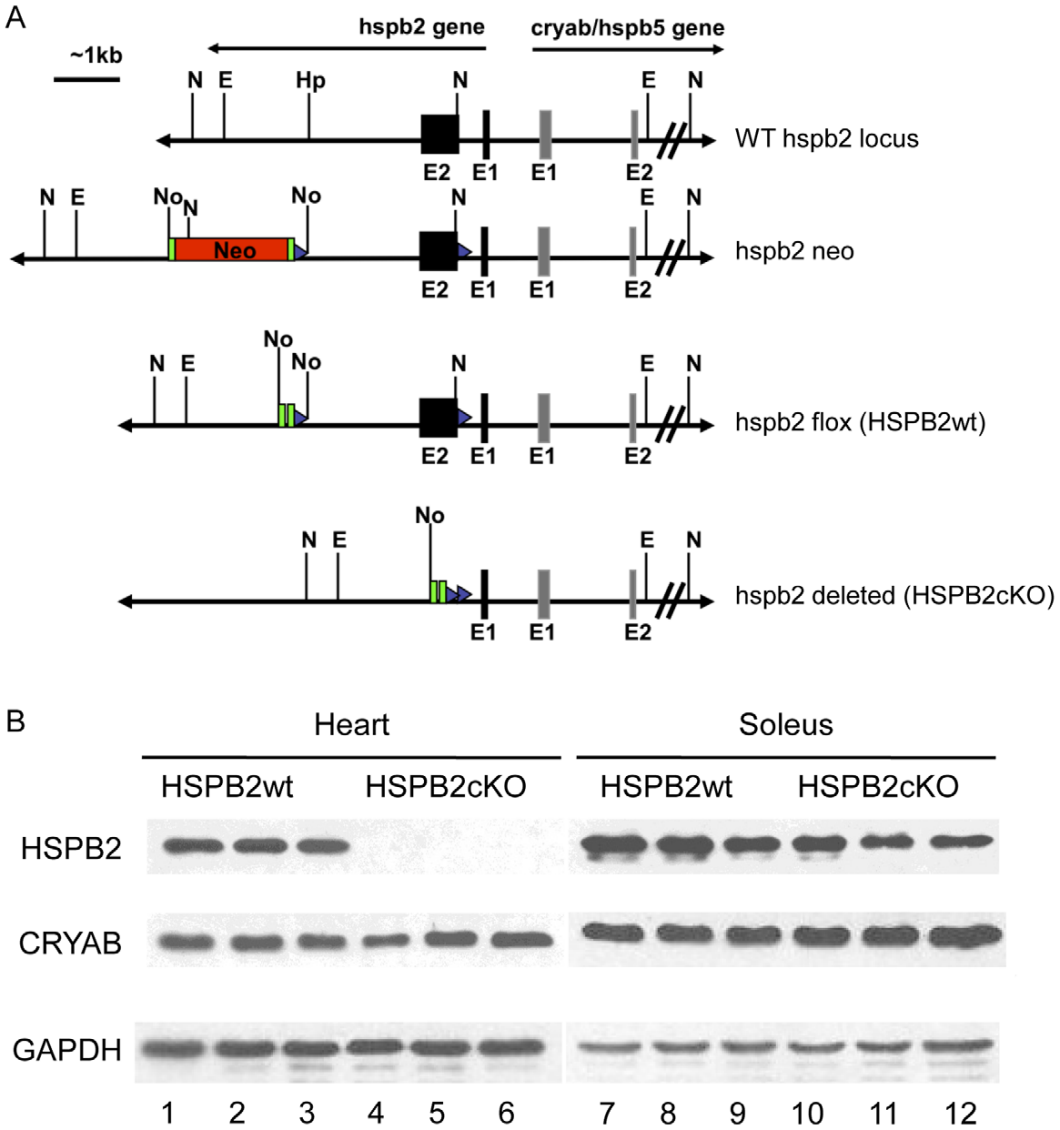
Generation of *hspb2* cardiac specific knockout mice. (A). Schematic diagram showing the *hspb2* locus, targeting *hspb2* construct and the expected organization of the targeted *hspB2* locus before and after Cre-mediated recombination. Abbreviations: N:NcoI, E:EcoRI, Hp:HpaI, No:NotI, Neo:neomycin cassette, E1: exon1, E2: exon2. Green rectangles represent FRT recombinase recognition sequences and blue triangles depict the loxP sites (Cre recombinase recognition sites). (B) Representative Western blots showing expression levels of the CRYAB and HSPB2 in hearts (lane 1–6) and soleus (lane 7–12). Heart extracts do not contain HSPB2 in HSPB2cKO (lane 4–6) while its expression is maintained in the skeletal muscle, soleus (lane 10–12). GAPDH was used a loading control.

The schematic structure of the wild type, targeted and deleted *hspb2* locus is depicted in Figure 1A. The 16 kb genomic DNA clone, encompassing *cryab* and *hspb2*, subcloned into pBluescript SK^-^ plasmid vector (pEW32) was a generous gift from Eric Wawrousek (NEI, NIH, Bethesda, MD, unpublished). Plasmid EW32 was digested with EcoRI and the ∼6.7 kb fragment containing the entire *hspb2* genomic region (exon I, intron I and exon II) with ∼3 kb of downstream sequence was isolated and cloned into pUC18 for subsequent restriction mapping and sequence analysis. The targeting vector was generated by incorporating a loxP oligonucleotide into the unique NcoI site in the first intron of the *hspb2* gene approximately 300 bp 3′ of exon 1. The second loxP site and neomycin gene cassettes (loxP-FRT-Neo-FRT) were inserted ∼1700 bp 3′ of exon 2 into a unique NotI site, created after NotI linker incorporation into the unique HpaI site, but in the same orientation as the first loxP site. By eliminating an essential domain for molecular chaperone activity, targeted deletion of the second exon containing the conserved α-crystallin domain by Cre recombinase of this ∼2600 bp fragment completely inactivates the functions of HSPB2. The targeting vector contains a 2.8 kb homologous region upstream of the first loxP site (long arm) and a 1.3 kb homologous region downstream of the loxP-FRT-Neo-FRT cassette (short arm). When crossed with FRT recombinase-expressing mice, the FRT sites flanking the neomycin gene cassette enabled the removal of this positive selection marker and, thus, any potential influences on the targeted or neighboring genes *in vivo*. The negative selection marker TK1 (thymidine kinase) was incorporated into the targeting vector at the 3′ end of the *hspb2* genomic sequence. Southern analyses of the NcoI digested ES cell genomic DNAs were performed with PCR probes generated outside of the borders of the ∼6.7 kb EcoRI fragment used for building the targeting construct to confirm homologous recombination (data not shown).

To eliminate the Neomycin cassette used for positive selection for the *hspb2* targeted ES cells, we obtained the FLP recombinase129S4/SvJaeSor-Gt(ROSA)26Sor^tm1(FLP1)Dym^/J mice that were crossed with the conditional *hspb2* targeted mouse line. *Hspb2* conditional targeted animals without the Neomycin cassette were crossed to cardiac specific (α-MHC) Cre expressor mice (CreTG) [18] to generate the *hspb2* knockout in the heart. Age matched male HSPB2wt and CreTG mice were used as controls.

### HSPB2 Antibody

Polyclonal antibodies raised against HSPB2 were generated for our laboratory by 21^st^ Century Biochemicals using the following peptide sequence: CPATAEYEFANPSRLGEQ-amide. This peptide was generated in-house and confirmed by mass spectrometry after HPLC purification. The peptide was injected with multiple protein carriers, using two rabbits. Serum was obtained after favorable titers were reached and HSPB2 antibody used in the present work was the affinity-purified fraction. The working dilution to probe western blots was 1∶5000.

### Western Blot Analysis

Hearts and soleus, slow-twitch skeletal muscle, from HSPB2cKO and HSPB2wt control mice were isolated, then washed several times in ice cold PBS to remove the blood. The tissue was minced into small pieces and homogenized in standard RIPA buffer. Supernatant was collected after cold centrifugation at 12000 g for 15 min. To prepare cytosolic/mitochondrial fraction, heart tissues were rinsed in cold PBS, homogenized in buffer containing 250 mM sucrose, 1 mM EGTA, 10 mM HEPES and 10 mM Tris-HCl (pH 7.4). The homogenates were centrifuged at 800 g for 7 min to pellet cell and tissue debris and the supernatants were further centrifuged at 4000 g for 15 min at 4°C. Those supernatants containing cytosolic fraction were saved for further use. The pellets containing mitochondria were thoroughly washed and centrifuged 2–3 times with the same buffer and then resolubilized in an EGTA free homogenization buffer. The suspensions were cleared using 0.1% NP40 and incubated for 30 min on ice [19]. Protein concentrations were estimated using the Bicinchoninic Acid assay kit (Thermo Scientific Cat No. 23227) and 20 µg of protein sample was used per lane for immunoblotting. Samples were boiled for 5 min in XT sample buffer (Biorad Cat No. 161-0791) and subjected to 10–12% SDS-PAGE. The proteins were transferred to nitrocellulose membrane and were probed with antibodies against HSPB2 (see description above), CRYAB/HSPB5 antibody (1∶5000) [20], HSP20 (HSPB6 (ADI-SPA-796, Enzo Life Sciences; 1∶2000), HSP25/HSPB1 (ADi-SPA- 801, Enzo Life Sciences; 1∶1000), VDAC (porin, PC548, Calbiochem: 1∶1000) and GAPDH (GAPDH -14C10, Cell signaling technology; 1∶5000). Appropriate horseradish peroxidase-conjugated secondary antibodies were used. The immunocomplexes were detected using enhanced pictogram chemiluminescence reagents (Thermoscientific Cat No. 1859674/75) and images obtained on X-ray film (Kodak) were scanned and quantitated by densitometric analysis using the ImageJ program (data not shown).

### RNA Analysis

Total RNA was extracted from ventricles in HSPB2wt and HSPB2cKO mice, using the RNeasy Mini Kit (QIAGEN). The cDNAs were prepared with the High Capacity cDNA Reverse Transcription Kit (Applied Biosystems). The accumulation of the PCR product using QuantiTect SYBR Green PCR Kit (QIAGEN) was monitored in real time by an ABI Prism 7900HT sequence detection system (Applied Biosystems). The following PCR primers (QIAGEN: hspb1: QT00094353; hspb2: QT00248220; hspb5: QT00094353; hspb6 QT00325738 and 18 S RNA: QT01036875 ) were used to amplifyhsp25/hspb1, hspb2, cryab/hspb5, hsp20/hspb6, and 18 S rRNA. The primer sequences for HIF1- α, IDH2, cox5b, CPT1-ß, MCAD, UCP2 are described elsewhere [21]. The results were analyzed with the ABI Prism 7900 HT system software (Applied Biosystems). The ABI Prism 7900 HT system records the number of PCR cycles (Ct) required to produce an amount of product equal to a constant threshold value, set to be reached during the exponential phase of the PCR reaction. Relative mRNA abundance was calculated from the real-time PCR data for the following experimental groups: 1) HSPB2wt (sham, n = 4), HSPB2wt (TAC, n = 4), HSPB2cKO (sham, n = 4), and HSPB2cKO (TAC, n = 3). All values were normalized with 18SRNA and presented, on the y-axis, as relative values to HSPB2wt (sham) set to 1.0.

### Transverse Aortic Constriction

TAC or sham surgery was performed as previously described [22] on 8 weeks old male CreTG, HSPB2wt, and HSPB2cKO mice. Briefly, after anesthesia with a single intraperitoneal injection of chloral hydrate (400 mg/kg), mice were placed in a supine position and a horizontal skin incision was made at the level of the suprasternal notch. The thyroid was retracted, and 2–3 mm longitudinal cut was made in the proximal portion of the sternum. A 6-0 silk suture was snared with the wire under the aorta between the origin of the right innominate and left common carotid arteries, and pulled back around the aorta. A bent needle was then placed next to the aortic arch, and the suture was tied around the needle and the aorta. A 27-gauge needle was used as the mode of moderate TAC, and a 30-gauge needle was used as the mode of severe TAC. After ligation, the needle was quickly removed. The sham procedure was identical except that the aorta was not ligated.

### Left Ventricular Catheterization and Pressure Measurements

At 4 weeks after moderate TAC and 8 weeks after severe TAC, LV pressure and its derivatives (+dP/dt and –dP/dt) were recorded using a Powerlab (ADInstrments, Colorado Springs, Colo) and catheter transducer inserted into the ascending aorta and LV chamber via the right common carotid artery.

### Protocols for Saponin-Permeabilized Fibers

Respiratory parameters of the total mitochondrial population were studied in situ in fresh saponin-permeabilized fibers as previously described [23]. Briefly, small pieces (2–5 mg) of cardiac muscle were taken from the left ventricle and permeabilized with 50 µg/ml saponin at 4°C in buffer A containing 7.23 mM K_2_EGTA, 2.77 mM K_2_CaEGTA, 6.56 mM MgCl_2_, 20 mM imidazole, 0.5 mM dithiothreitol, 53.3 mM K-methanS, 20 mM taurine, 5.3 mM Na_2_ATP, 15 mM PCr and 3 mM KH_2_PO_4_, pH 7.1 adjusted at 25°C. The fibers were then washed twice for 10 min in buffer B containing 7.23 mM K_2_EGTA, 2.77 mM K_2_CaEGTA, 1.38 mM MgCl_2_, 20 mM imidazole, 0.5 mM dithiothreitol, 100 mM K-methanS, 20 mM taurine, 3 mM KH_2_PO_4_ and 2 mg/ml BSA, pH 7.1 at 25°C.

### Fiber Respiration and ATP Measurements

The respiratory rates of saponin-permeabilized cardiac fibers were determined using an oxygen sensor probe in 2 ml buffer B at 25°C with continuous stirring. Studies were performed with 0.02 mM palmitoyl-carnitine and 2 mM malate as substrates [24]. Oxygen consumption rates are expressed in nmol O_2_ min/mg dry fiber weight. State 2 respiration (no ADP) was measured in a 125 mM KCl solution containing the appropriate substrates. State 3 (ADP-dependent) respiration was measured by adding ADP at a final concentration of 1 mM, which stimulates maximum activation of respiration. State 4 respiratory rates were measured by the addition of 1 µg/mL of oligomycin, an inhibitor of ATP synthase. ATP concentration was determined by a bioluminescence assay based on the luciferin/luciferase reaction using the ATP assay kit ((Enliten ATP Assay Kit, Promega, Madison, WI). The mitochondrial analysis was performed in HSPB2wt and HSPB2cKO mice at 4 weeks after moderate TAC and compared with sham-operated mice.

### Statistical Analysis

All data are presented as mean ±SD. Significance levels were analyzed by Student’s t-test or one factor analysis of variance (ANOVA) followed by Tukey post-hoc test comparison of means, using Prism (GraphPad, San Diego, CA). A value of P<0.05 was considered statistically significant.

**Figure 2:**
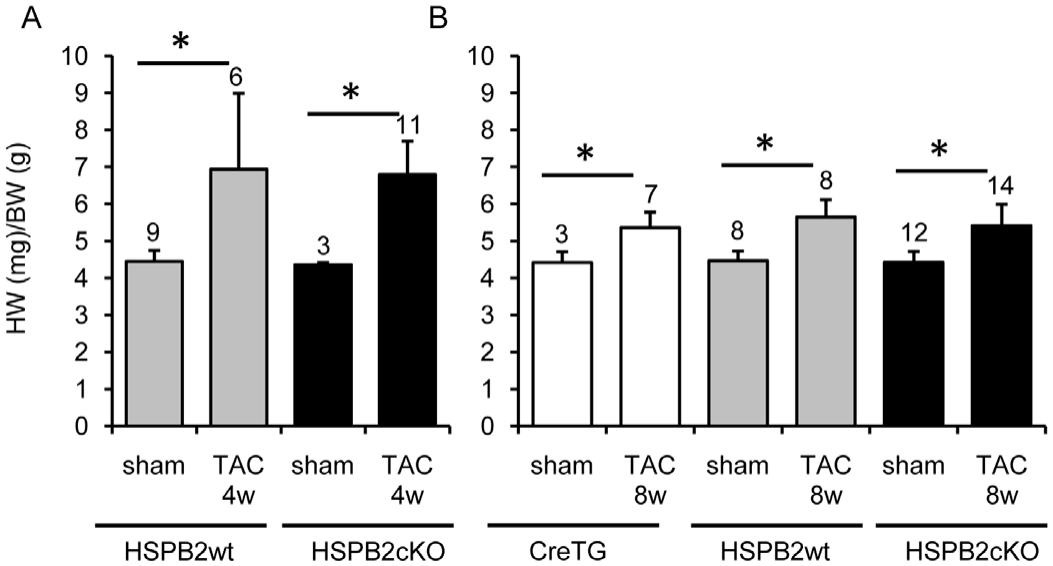
Loss of HSPB2 expression does not alter the cardiac hypertrophic response to transaortic constriction (TAC). Heart weight (HW) and body weight (BW) were measured at four weeks after TAC in HSPB2wt (grey bars) and HSPB2cKO (black bars) (A) and at eight weeks after TAC in CreTG (white bars), HSPB2wt (grey bars) and HSPB2cKO (black bars) (B). TAC induced significant cardiac hypertrophy in all experimental animals in comparison to sham-operated ones.*P<0.05 compared with each sham group. No difference was observed between the HSPB2 cardiac deficient animals and those expressing HSPB2. The number of animals analyzed in each group is indicated above the bars.

## Results

### Cardiac Specific Disruption of the hspb2gene in Mice

Cardiac specific disruption of the *hspb2* gene was obtained by using a floxed allele introduced by homologous recombination and the cardiac expression of Cre recombinase driven by αMHC promoter (see details in Materials and Methods) (Figure 1A). Mouse breeders, which were expected to provide HSPB2cKO gave the anticipated number of pups, indicating that there was no embryonic lethality related to the lack of HSPB2 expression (data not shown). Western blot analysis (Figure 1B) showed that heart tissue from HSPB2cKO mice expressed CRYAB (HSPB5) in contrast to the DKO previously reported, and did not contain any HSPB2. Skeletal muscle (soleus), which was not targeted for recombination in HSPB2cKO, expressed both CRYAB (HSPB5) and HSPB2, confirming that we had successfully produced cardiac specific HSPB2 deficient mice (Figure 1B). There was no change of the expression level of CRYAB between HSPB2cKO and HSPB2wt hearts.

**Figure 3:**
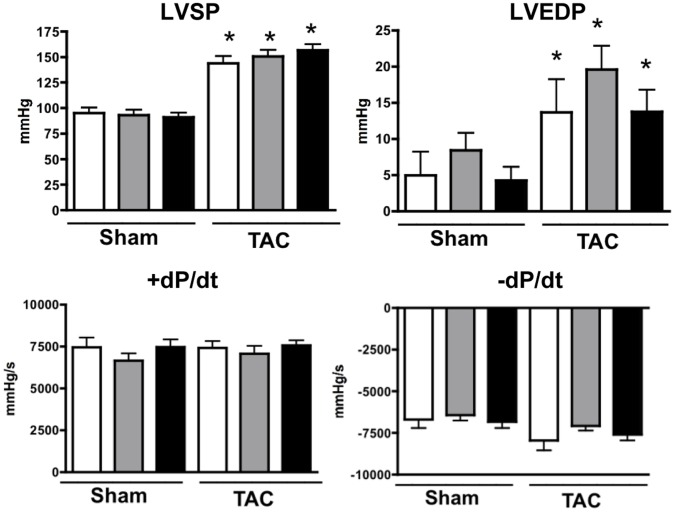
Loss of HSPB2 expression does not modify LV function in response to transaortic constriction (TAC). Changes in LV systolic peak pressure (LVSP), end-diastolic pressure (EDP), maximal rate of LV pressure rise (+dP/dt), maximal rate of LV pressure fall (−dP/dt) were measured in sham and TAC operated animals from the three genotypes: CreTG (white bars), HSPB2wt (grey bars) and HSPB2cKO (black bars). There were no significant differences of any hemodynamic indexes between HSPB2cKO and control hearts.*P<0.05 compared with each sham group. Four to eleven animals were analyzed for each group.

As a part of our initial characterization of the HSPB2cKO mice, a longevity study was performed. Through 1 year of age, only one of 14 HSPB2wt mice (93% survival) and one of 15 HSPB2cKO mice (93% survival) died. Thus, there was no significant difference of survival between HSPB2cKO and HSPB2wt mice. Also, heart weight (HW) and HW/body weight (BW) ratios were not significantly different between 1-year-old HSPB2wt (BW 29.68±5.18 g, HW 140.7±22.2 mg, HW/BW = 4.77±0.43) and HSPB2cKO mice (BW 31.25±5.07 g, HW 135.9±23.7 mg, HW/BW = 4.36±0.47).

**Figure 4:**
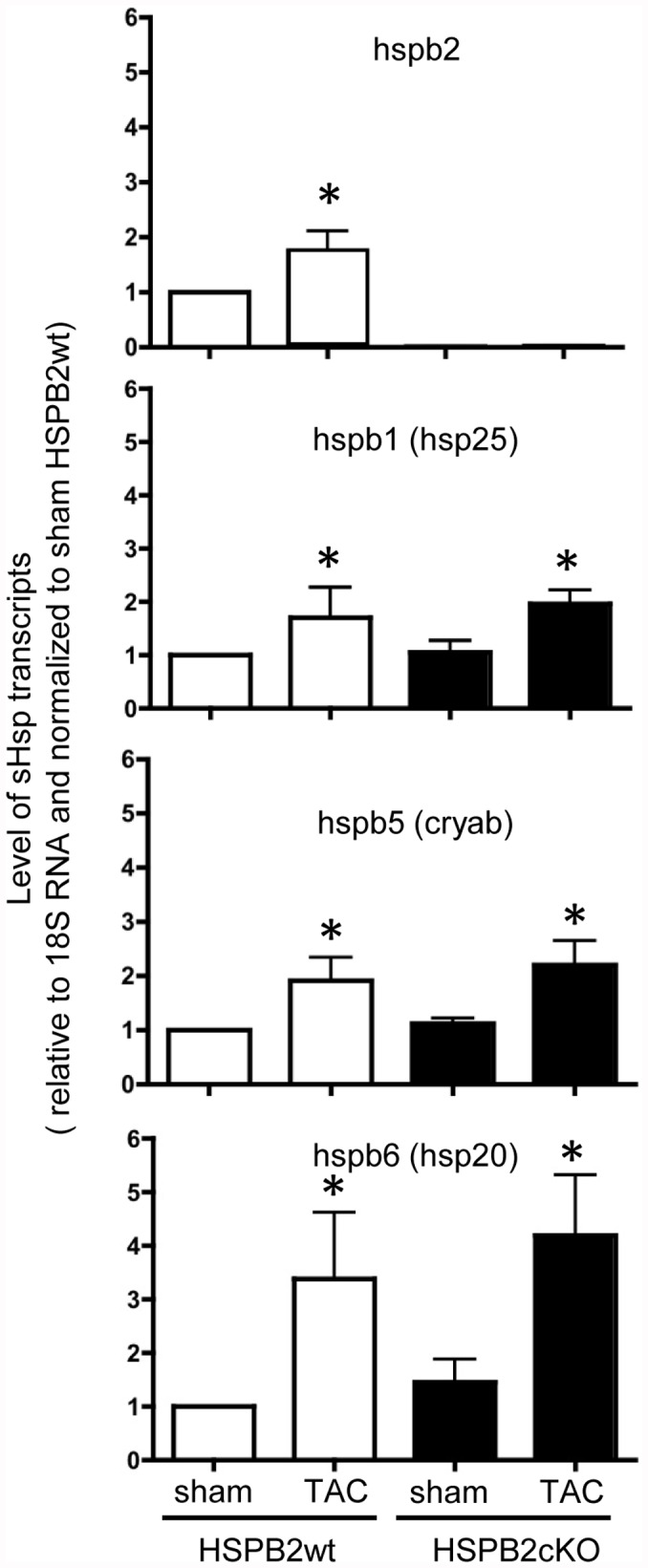
Small Hsp transcript levels measured in hearts from sham and TAC- operated animals by quantitative RT-PCR analysis. Transcript levels were measured for hspb2, hspb1, hspb5, hspb6 in HSPB2wt and HSPB2cKO animals, after sham or TAC-surgery. The transcript abundance increased significantly in response to pressure-overload. Except for hspb2, there were no significant differences between sHsp mRNA expression in HSPB2wt and HSPB2cKO hearts in both of sham and TAC operated mice. From 3 to 5 heart samples were analyzed per group. *P<0.05compared with each sham groups.

### Pressure Overload-induced Cardiac Hypertrophy

Because HSPB2 is dispensable for cardiac function under normal/physiologic conditions, we investigated the hypertrophic response of HSPB2 deficient hearts under pressure overload induced by transaortic constriction (TAC). At 4 weeks after moderate TAC, cardiac hypertrophy (Figure 2A) and LV function between HSPB2cKO and HSPB2wt hearts were similarly modified (data not shown). To increase the level of cardiac stress, a more severe TAC procedure was applied and the animals were characterized 8 weeks after surgery. Even with this augmented level of pressure overload stimuli for longer duration, HSPB2wt and HSPB2cKO did not show any significant differences in lethality. We found 2 of 19 (10%) versus2 of 21 (10%) deaths between the HSPB2wt and HSPB2cKO groups, respectively. Similar findings were observed with 1/12 (8%) in α-MHC Cre (i.e., named CreTG) mice. Notwithstanding, the HW/BW ratio calculated at 8 weeks after surgery was significantly increased in TAC versus sham-operated mice (>20% increase, P<0.05) but there was no significant difference in the hypertrophic response exhibited among these three groups of mice (CreTG, HSPB2wt and HSPB2cKO) (Figure 2B).

**Figure 5:**
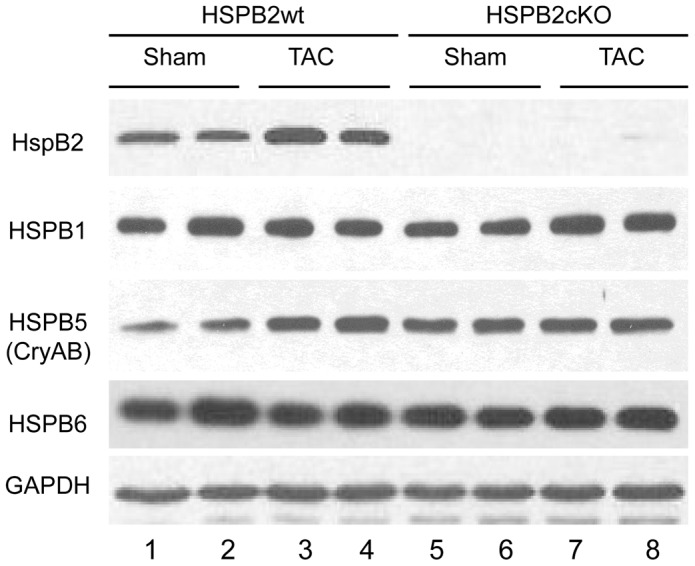
No significant changes in expression of small HSP in the absence of HSPB2 and/or under cardiac stress conditions. Hearts from HSPB2wt and HSPB2cKO were isolated at 8 weeks after sham or TAC procedure and protein extracts (soluble fraction) were analyzed by western blots probed with anti-HSPB2, HSPB1 (HSP25), HSPB5 (CRYAB) and HSPB6 (HSP20). In contrast to transcripts, protein levels were not significantly modified in samples from TAC operated mice and except for HSPB2, there was no difference between HSPB2cKO and HSPB2wt samples. Two to five samples were analyzed per group and a representative example is shown.

Parameters for cardiac hemodynamics, which were evaluated at 8 weeks after TAC, did reflect the cardiac response to pressure overload but again did not reveal any significant variation among the three groups of mice (CreTG, HSPB2wt and HSPB2cKO). The mean values for left ventricular systolic pressure (LVSP) and end-diastolic pressure (LVEDP) are graphically presented with +dP/dt, and –dP/dt in Figure 3. Lung weight (LW)/BW ratio at 8 weeks after severe TAC was comparable between sham and TAC operated mice in all three groups, indicating that TAC-operated mice were not affected by pulmonary edema, a well-established clinical determinant of left ventricular failure (data not shown).

**Figure 6:**
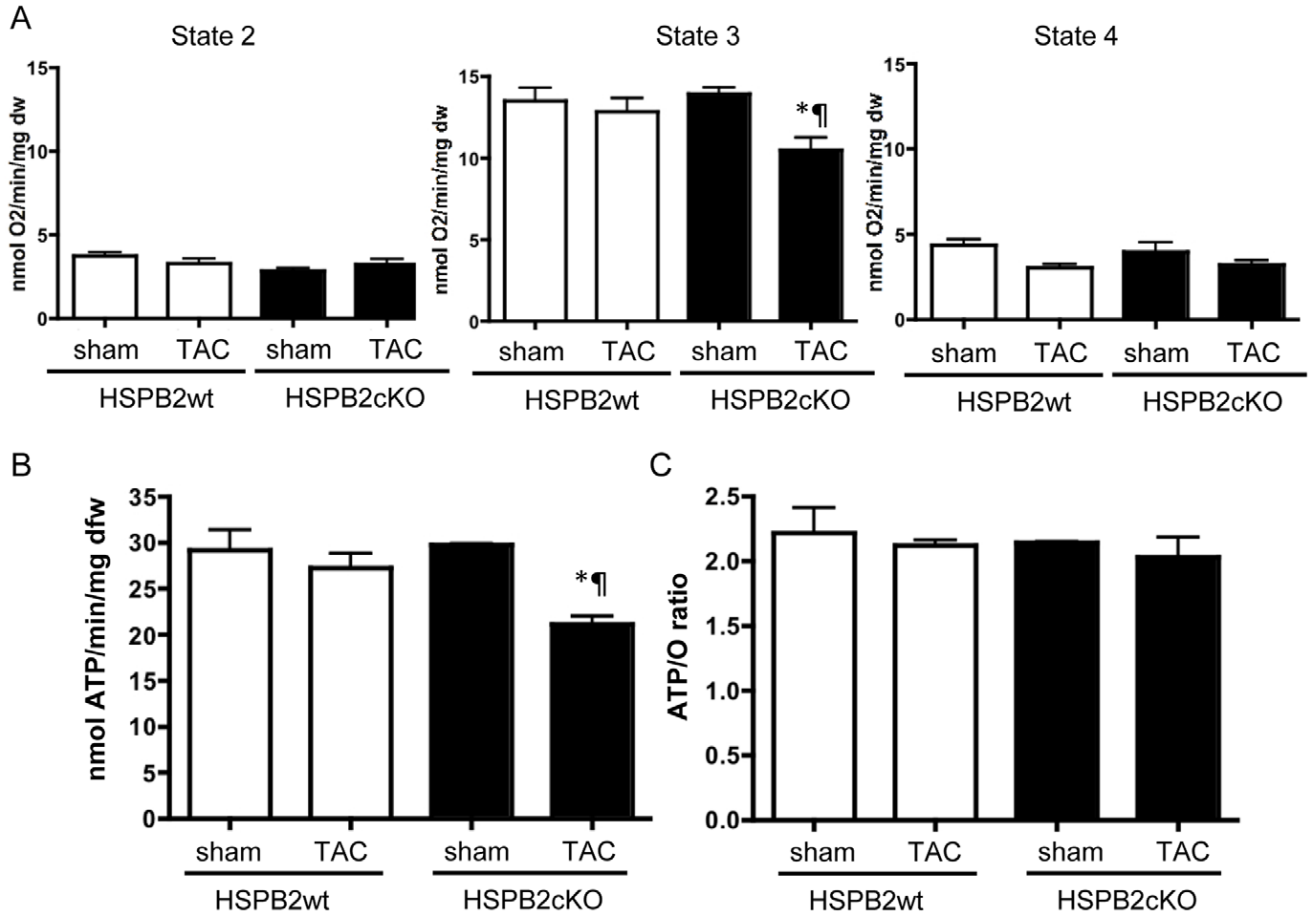
Mitochondrial function is altered in HSPB2cKO hearts under cardiac stress. (A) Mitochondrial respiration was measured in saponin-permeabilized cardiac fibers from HSPB2wt (sham; n = 16, TAC; n = 8) and HSPB2cKO left ventricle (sham; n = 4, TAC; n = 8) at 4 weeks after sham and TAC procedure. State2 corresponds to respiration in the absence of ADP; state 3 represents ADP-stimulated respiration and state 4 to oligomycin-inhibited respiration. State 3-respiration rate in TAC operated HSPB2cKO mice was significantly reduced compared with that in sham HSPB2cKO operated mice (P<0.05). (B) ATP production rate and (C) ATP-to-O ratios (ATP/O) were obtained from permeabilized fibers, where O refers to oxygen consumption under state 3 conditions. ATP production in TAC operated HSPB2cKO mice was significantly reduced compared with that in sham operated HSPB2cKO mice and TAC operated HSPB2wt mice (P<0.05). There was no significant difference of ATP/O between HSPB2wt and HSPB2cKO hearts in both of sham and TAC operated mice. *P<0.05compared with each sham groups. P<0.05 compared with HSPB2wt animals.

### sHSP Expression in Response to HSPB2 Deficiency and Cardiac Hypertrophy

The small HSPs form a multi protein family whose members share partially overlapping profiles of expression, molecular structure and interactions [1], [2], [3]. Therefore, it is conceivable that *hspb2* deletion might be compensated by changes in expression of other members of the small HSP family. Furthermore, the imposed cardiac stress could trigger additional modifications in sHSP expression.

**Figure 7:**
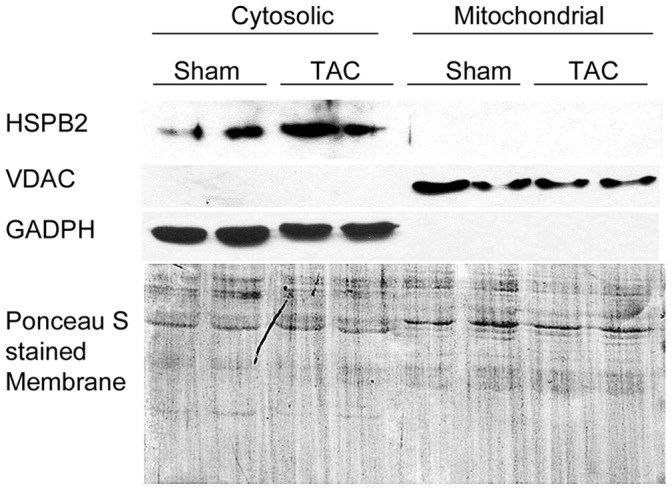
Endogenous HSPB2 does not exhibit cytosolic to mitochondrial translocation upon cardiac pressure overload stress. Cytosolic and mitochondrial fractions were prepared from HSPB2wt mouse hearts collected 8 weeks after sham or TAC surgery (see Materials &Methods). The western blot was probed with HSPB2, GAPDH and VDAC antibodies. GADPH and VDAC are markers used to validate the separation of cytosolic and mitochondrial fraction, respectively. Ponceau S staining was performed to demonstrate the equal loading.

To test these hypotheses, we first measured the relative abundance of sHSP transcripts by RT-qPCR. TAC operated hearts exhibited an increased transcript levels of the four sHSPs tested (hspb1 mRNA: 1.7±.0.9 fold; hspb2 mRNA:1.8±0.6 fold; hspb5 mRNA: 1.9±0.7, hspb6 mRNA: 3.4±2.1 fold) (Figure 4). Hspb2 deficiency did not change the transcript level of hspB1, hspb5 and hspb6 under normal conditions or after TAC (Figure 4).

In contrast to the marked increased in transcripts, the protein levels of HSP25 (HSPB1), HSPB2, CRYAB (HSPB5), HSP20 (HSPB6) insoluble protein extracts from hearts at 8 weeks after sham and TAC procedure were not significantly modified (Figure 5).

**Figure 8:**
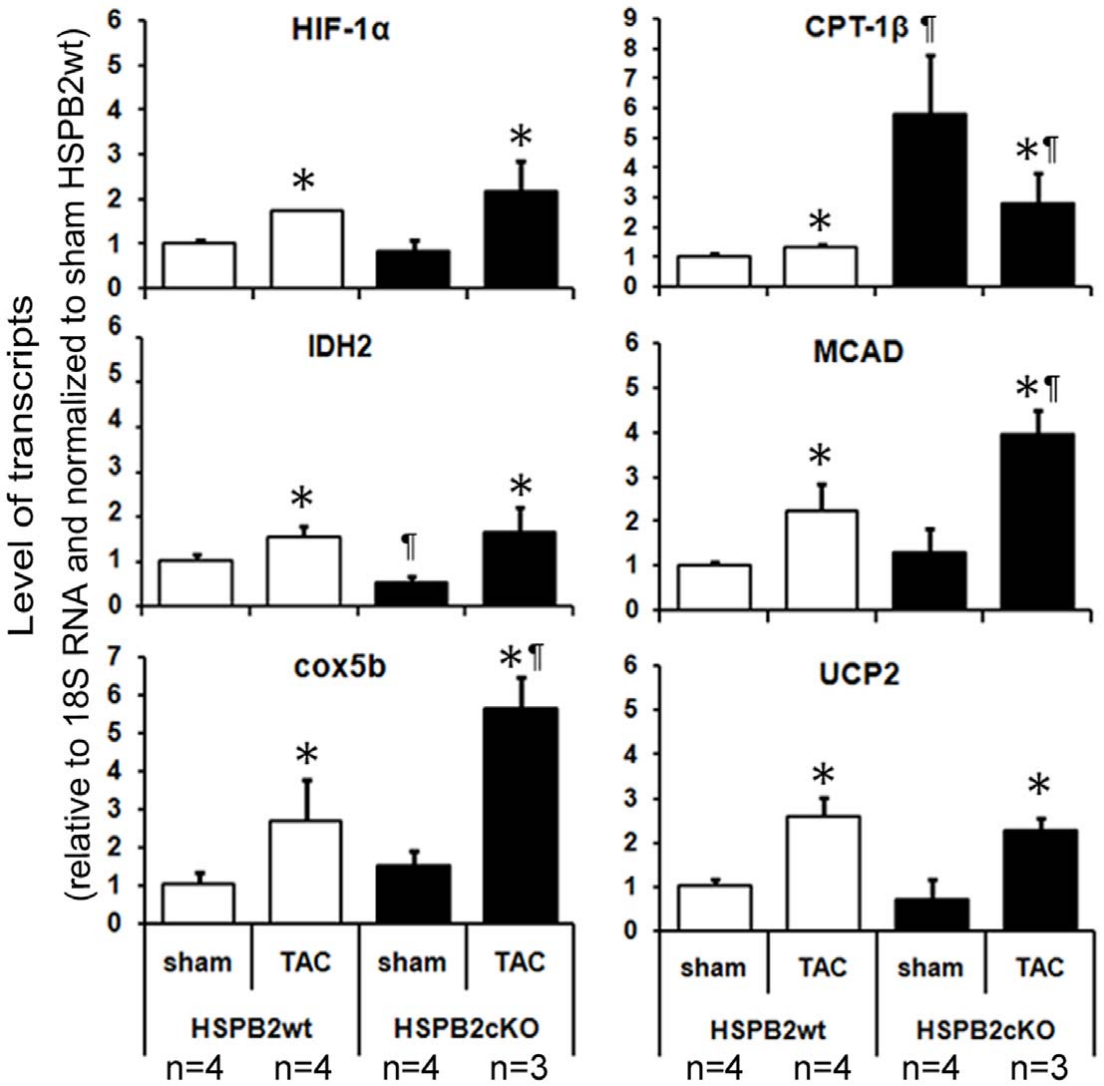
Representative expression of genes involved in mitochondrial metabolism between HSPB2wt and HSPB2cKO hearts after pressure overload conditions. RT-qPCR was used to analyze transcript levels in mouse hearts collected 8 weeks after sham or TAC surgery. Shown here are hypoxia-inducible factor 1, alpha subunit (HIF-1α), and isocitrate dehydrognease 2 (IDH2) are involved in glycolysis; cytochrome c oxidase subunit 5B (cox5b) is involved in oxidative phosphorylation (OXPHOS); carnitine palmitoyltransferases (CPT-1β), medium-chain acyl-CoA dehydrogenase (MCAD) and mitochondrial uncoupling protein 2 (UCP2) are involved in fatty acid oxidation (FAO). *P<0.05 compared with each sham groups. P<0.05 compared with HSPB2wt animals.

### Mitochondrial Fiber Respiration and ATP Production

Previously, we had shown that a mouse model deficient in HSPB2 but overexpressing CryAB had slower and less complete recovery of cardiac energetics during reperfusion after ischemia, and that this metabolic defect was similar in *Hspb2.5^−/−^* double knockout animals [12]. Those data had suggested that HSPB2 could be involved in mitochondrial function [12]. As this study was performed with DKO mice, which partially rescued by a transgene overexpressing CRYAB (HSPB5), we sought to unequivocally clarify whether or not HSPB2 expression plays a critical role in mitochondrial function using our newly created HSPB2cKO mice.

We assessed mitochondrial fiber respiration rate and ATP production both under physiological conditions and under pathological conditions of cardiac hypertrophy induced by pressure overload. The mitochondrial respiration rates, using palmitoyl-carnitine and malate as substrate, were indistinguishable between HSPB2wt and HSPB2cKO under normal basal conditions (data not shown), whereas state 3 respiration rates (ADP stimulated) in HSPB2cKO was significantly reduced in TAC-compared to sham operated mice (P<0.05) (Figure 6A). Also state 4-respiration rate showed a tendency to be lower in HSPB2cKO and HSPB2wt TAC operated mice versus sham operated ones (P = 0.06). In addition, ATP production in TAC operated HSPB2cKO mice was significantly reduced compared with that in sham operated HSPB2cKO mice and TAC operated HSPB2wt control mice (P<0.05) (Figure 6B). There was no significant difference of ATP/O between HSPB2wt and HSPB2cKO hearts in both of sham- and TAC operated mice (Figure 6C), indicating that mitochondrial ADP stimulated respiration rates and ATP production in HSPB2cKO hearts were proportionally reduced under conditions of cardiac hypertrophy.

### HSPB2 Subcellular Localization

Because mitochondrial state 3 respiration was significantly different in stressed heart lacking HSPB2, we wanted to test the hypothesis that TAC procedure was triggering HSPB2 translocation to mitochondria. This hypothesis was supported by previous data showing that C2C12 cells transfected with recombinant HspB2 construct displayed such association between HSPB2 and mitochondria [16].

Taking advantage of the new HSPB2 antibody we had generated, we performed western blots to detect HSPB2 in cytosolic and mitochondrial fractions prepared from HSPB2wt hearts collected 8 weeks after TAC. Under these conditions, we found HSPB2 was only detected in the cytosolic but not mitochondrial fraction, suggesting that cytosolic HSPB2 localization remains unchanged after pressure overload stress *in vivo* (Figure 7). To determine whether the translocation of HSPB2 occurs transiently after acute pressure overload, we performed a similar experiment using samples collected only 3 days after TAC, which also revealed HSPB2 remains exclusively in the cytosolic fraction (data not shown). We confirmed by confocal microscopy, using paraffin sections from HSPB2wt and HSPB2cKO, the absence of dynamic changes in the subcellular localization of HSPB2 at the Z-band, following either sham or TAC operations (data not shown). Taken together, the subcellular localization of HSPB2 does not appear to be significantly altered by cardiac stress after pressure overload in the intact murine heart.

### Alteration of Metabolic Regulators by HSPB2 Deficiency

Cardiac stress such as pressure overload modifies expression of genes encoding certain metabolic regulators. We, therefore, hypothesized that HSPB2 could indirectly impact the response of metabolic regulators in response to pressure overload. To test this hypothesis, we arbitrarily selected for further analysis several genes involved in glucose metabolism such as hypoxia-inducible factor1, alpha subunit (HIF-1α) and isocitrate dehydrogenase 2 (IDH2, mitochondrial), OXPHOS such as cytochrome C oxidase subunit b(COX5b), in fatty acid oxidation such as carnithine palmitoyltransferase 1 β (CPT-1β), acyl-coenzyme A dehydrogenase medium chain (MCAD, Acadm) and uncoupling protein 2 (UCP2, mitochondrial proton carrier) [21]. Following reverse transcription with quantitative PCR, we determined the relative changes in transcript levels among sham- and TAC-operated samples from both genotypes, HSPB2wt and HSPB2cKO. All experiments were performed in triplicate usually with 3 or 4 animals per experimental group. Among the representative genes contained in this limited survey, the majority of transcripts (i.e., HIF-1α, IDH2, cox5b, MCAD and UCP2) were modestly increased by TAC but comparable for both HSPB2wt and HSPB2cKO under these experimental conditions (Figure 8). In contrast, basal levels of CPT-1β transcript were significantly elevated and were down regulated in HSPB2cKO compared with HSPB2wt by TAC after 8 weeks. Taken together, our findings indicate for the first time that chaperone HSPB2 expression has hitherto unspecified effects on the expression levels of several metabolic regulators under basal and stress-inducible conditions in vivo.

## Discussion

In this study, we describe a new mouse model characterized by the cardiac specific deletion of the *hspb2* gene (HSPB2cKO). Under normal conditions, HSPB2cKO did not exhibit any obvious cardiac anomaly and this is consistent with the observations made with the DKO animals, which were deficient in both *hspb2* and *hspb5* [8]. We further demonstrated that lack of HSPB2 did not modify the cardiac response to pressure overload in response to either mild or severe stress. Because the expression levels for some other sHSPs such as HSPB1, HSPB5 and HSPB6 were not visibly altered, absence of phenotype in HSPB2cKO does not seem to be linked to major compensatory responses. As the present study analyzes a cardiac-specific knockout of *hspb2*, we could not definitively address the role of HSPB2 deficiency in other organs especially in skeletal muscles that harbor high levels of HSPB2 expression.

The superfamily of small MW HSPs (∼18 to 32 kDA) has been implicated in diverse functions and biological roles ranging from cellular immunity to oncogenesis to cardiomyopathy and heart failure [25], [26]. Whereas both cryab and hspb2 are arranged adjacently in the genome, we have hypothesized the existence of tissue-specific functions for their expression, under the control of myogenic regulators that drive high levels of endogenous expression in skeletal muscle and the heart. Unlike distinct mutations in human CryAB that have been linked to various inherited multisystem diseases (see references in [27]), HSPB2 is not only the most divergent sHSP family (i.e., 30% sequence identity to all other mammalian sHSPs) but its biological function remains unknown. We confirm that HSPB2, like CryAB, is dispensable for cardiac function and maintenance of myocardial integrity. Similarly the knockout of *hspb1*, another sHSP highly expressed during heart development did not provoke any major disturbance in cardiac anatomy or function as evidenced by the normal lifespan of those animals [28].

Although prior studies by other investigators have shown that isolated and intact hearts lacking both CRYAB and HSPB2 (DKO) exhibit severe contractile dysfunction and increased myocardial injury in response to ischemia/reperfusion *ex vivo* [9], [11], our laboratory has reported increased resistance of DKO hearts to *in situ* and *ex* *vivo* ischemic conditions compared with wild-type controls [12], [17]. Such studies, therefore, have provided confounding insights about the functional roles of these two sHSPs in ischemic cardioprotection and in no study could the specific role of HSPB2, in particular, be unambiguously assigned. In addition, we found that HSPB2 appears to be required for systolic performance and for maintaining cardiac energetics in the isolated perfused mouse heart. To address these unmet needs, we have advocated the use of genetic tools to unmask potential novel and non-redundant functions between CryAB and HSPB2 in terms of cardiac mechanics and energetics.

Based on an earlier report on co-localization of HSPB2 [16], our laboratory had subsequently implicated HSPB2 functions to mitochondrial bioenergetics pathways. The mitochondrial respiration rates and ATP production in DKO mice were reduced compared with those in control mice [17]. Pinz et al reported that HSPB2, independent and distinct from CRYAB, was required for efficient coupling of the free energy of ATP hydrolysis and contractile performance [12]. The present study demonstrated that the mitochondrial respiration rates and ATP production were indistinguishable between HSPB2cKO and control under basal conditions, whereas ADP stimulated respiration rates and ATP production in TAC operated HSPB2cKO hearts was lower than that of TAC operated control hearts, indicating that mitochondrial fatty acid beta oxidation and ATP production were depressed in HSPB2cKO hearts following cardiac pressure overload. Although it has been reported that mitochondrial dysfunction may develop prior to or in parallel with the onset of systolic dysfunction in pathological cardiac hypertrophy [29], other studies have shown that modest (<30%) impairment of mitochondrial dysfunction is well compensated within the functional contractile reservoir without modifications of cardiac hemodynamic parameters [21], [24], [30]. This outcome is in agreement with our findings, which measured a similar cardiac systolic function between HSPB2cKO and HSPB2wt mice under pressure overload conditions.

How might HSPB2 expression modulate cellular metabolism and mitochondrial function under cardiac stressful conditions without evidence for direct interactions between the chaperone and organelle? The basic mechanism(s) for these intriguing observations are presently unknown but our preliminary studies hint at possible effects of HSPB2 expression on several metabolic regulators. For example, we found that HSPB2 deficiency was associated with up-regulation of CPT-1ß under basal conditions but, unexpectedly, CPT-1ß was down-regulated under cardiac stress using TAC [29], implicating a key role of HSPB2 for FAO since HSPB2wt compared with HSPB2cKO was entirely unaffected by TAC. Of interest, the chaperone-like activities of HSPB2 have been shown recently to specifically interact with some client proteins (e.g. insulin, alcohol dehydrogenase) but not others (e.g. citrate synthase) [31]. Altogether, these findings provide a compelling rationale of future studies to define the complete interactome of sHSPs such as HSPB2 in cardiac cells–under both basal and stress conditions.

Although the first description of the gene encoding HSPB2 expression was reported in 1998 by Suzuki and collaborators [5], HSPB2 remains a novel chaperone in cardiac biology and physiological especially in relation to certain non-redundant functions not specified by additional member of the multigene sHSP family [31]. Our new mouse model provides a valuable tool to further such investigations as well as to consider additional roles of HSPB2, besides the striated and smooth muscle lineages.
